# Transitioning a home telehealth project into a sustainable, large-scale service: a qualitative study

**DOI:** 10.1186/s12913-016-1436-0

**Published:** 2016-05-16

**Authors:** Victoria A. Wade, Alan D. Taylor, Michael R. Kidd, Colin Carati

**Affiliations:** Discipline of General Practice, The University of Adelaide, North Tce., Adelaide, 5005 Australia; Faculty of Medicine, Nursing and Health Sciences, Flinders University, Adelaide, 5042 Australia

**Keywords:** Home telehealth, Sustainability, Qualitative study, Deliberative forum, Change management, Models of care

## Abstract

**Background:**

This study was a component of the Flinders Telehealth in the Home project, which tested adding home telehealth to existing rehabilitation, palliative care and geriatric outreach services. Due to the known difficulty of transitioning telehealth projects services, a qualitative study was conducted to produce a preferred implementation approach for sustainable and large-scale operations, and a process model that offers practical advice for achieving this goal.

**Methods:**

Initially, semi-structured interviews were conducted with senior clinicians, health service managers and policy makers, and a thematic analysis of the interview transcripts was undertaken to identify the range of options for ongoing operations, plus the factors affecting sustainability. Subsequently, the interviewees and other decision makers attended a deliberative forum in which participants were asked to select a preferred model for future implementation. Finally, all data from the study was synthesised by the researchers to produce a process model.

**Results:**

19 interviews with senior clinicians, managers, and service development staff were conducted, finding strong support for home telehealth but a wide diversity of views on governance, models of clinical care, technical infrastructure operations, and data management. The deliberative forum worked through these options and recommended a collaborative consortium approach for large-scale implementation. The process model proposes that the key factor for large-scale implementation is leadership support, which is enabled by 1) showing solutions to the problems of service demand, budgetary pressure and the relationship between hospital and primary care, 2) demonstrating how home telehealth aligns with health service policies, and 3) achieving clinician acceptance through providing evidence of benefit and developing new models of clinical care. Two key actions to enable change were marketing telehealth to patients, clinicians and policy-makers, and building a community of practice.

**Conclusions:**

The implementation of home telehealth services is still in an early stage. Change agents and a community of practice can contribute by marketing telehealth, demonstrating policy alignment and providing potential solutions for difficult health services problems. This should assist health leaders to move from trials to large-scale services.

## Background

Telehealth can be used for the delivery of specialist services to the home, such as rehabilitation [1] or palliative care [2], which would otherwise be provided in the hospital or by in-person home visits. The Flinders University Telehealth in the Home: Palliative and Aged Care trial (FTH trial) added home telehealth to existing in-person specialist outreach services. The FTH trial operated for 18 months in the southern Adelaide area in 2013–14, and developed telehealth models of care for rehabilitation, palliative care, and aged care. For rehabilitation and palliative care services, patients received video consultations from medical, nursing and allied health staff, monitoring devices for physical activity and weight, and self-assessment applications for status reporting. In aged care, the FTH trial provided video assessments from specialist geriatricians to residents of aged care facilities [3, 4].

Evaluation found high acceptance from patients and providers, substantial reductions in staff travel time, more timely clinical intervention for palliative care patients, and that more allied health services could be delivered to rehabilitation patients in the home, with positive functional outcomes. For aged care, access to specialist assessment was provided where this was previously unavailable, and changes were made to medication and management plans for the majority of patients. Subsequently, each of the three clinical services indicated that they wished to consider how these services could be expanded and continued as part of routine service delivery after the trial concluded.

The difficulty of achieving both sustainable and large-scale telehealth services are known problems that have been reported in the research literature for over 20 years [5, 6]. Previous research into the uptake and sustainability of telehealth has identified the major enablers of usable technology, planned organisational change, provider acceptance, provider collaboration, the adoption of a business model, and policy support [7–9]. The relative importance of each enabler and the relationships between them is not well understood: a review of the introduction of technology into healthcare notes the absence of simple and useful models [10], while two other reports [11, 12] conclude that complex relationships between the technical, social and organisational dimensions need to be negotiated to achieve implementation, but that these processes have received scant attention in research [11].

**Fig. 1:**
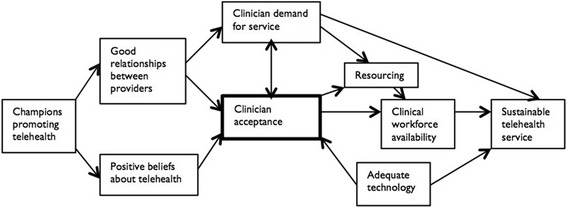
Model of Telehealth Service Sustainability

The first author has been conducting a program of investigation into these issues, defining the three main phases of telehealth implementation as initiation, sustainability, and large-scale uptake. A study of 36 Australian telehealth services, using a grounded theory approach, concluded that champions are the main drivers of initial implementation [13], and that clinician acceptance is the key factor for making a successful transition to sustainable operations [14], where sustainability is defined as the ability to continue operating into the future without obvious threats [15]. The model for sustainable operations is shown in Fig. 1. At the time, this was as far as the model could be developed because all the services in the sample were small-scale, and the grounded theory could only reflect the context from which it was constructed.

The remaining challenge, therefore, is to understand how to scale up telehealth into routine use within the broader healthcare system. A large cluster RCT in the United Kingdom has found that attempts to introduce such a service for home telehealth can fail if implementation is too rigid and not related to existing primary care services [16]. By contrast, the Veterans Health Administration in the USA is a successful large-scale telehealth system that was built up by commencing with local innovation, then creating overarching clinical, technical and business systems that supported implementation at multiple sites [17].

This study was commissioned by the FTH trial to answer this research question: “How can large-scale implementation of home telehealth be achieved in South Australia?”. The study consulted both individually and collectively with individuals who had expertise, influence, or direct responsibility for developing home telehealth services, and used qualitative methods to produce: 1) a preferred implementation approach, and 2) a process model that offers practical guidance for achieving this goal.

## Methods

Action research, which has been identified as particularly useful in researching the diffusion of healthcare innovations [18], was used as the methodology for conducting the research, and grounded theory was used as the theoretical framework to construct the process model [19].

The first phase of consultation consisted of semi-structured interviews with senior clinicians, health service managers, and policy makers. The interviewees were selected by having a connection to home telehealth (including but not necessarily the FTH Trial), enough seniority to have influence over transitioning the FTH Trial into a sustainable service, and to have a diversity of roles. To understand broader scale uptake it was considered necessary to engage with those in the wider healthcare system, so interviewees were recruited both from those who conducted the FTH trial, and those who had an interest in the area but were not directly involved. Recruitment occurred by email, followed up by a telephone call if willingness to continue was indicated. Written consent was obtained from each participant. The maximum number of interviewees was set in the mid-twenties, to ensure a manageable number for a later consultative forum, but could be ceased earlier if data saturation was reached.

The interviewees were asked about models for future implementation of telehealth in the home services, posed questions about how telehealth service development should proceed, and asked to respond to a list of issues affecting telehealth. The questions were selected according to the participant’s area of expertise, and the interview schedules are given in the Appendix. The interviews were audio recorded, transcribed, and entered into NVivo software for data handling [20]. After open coding, a thematic analysis was conducted to identify factors that were relevant to achieving a sustainable, large-scale service, and to develop a small number of options for future implementation.

Subsequently, the interviewees and other decision makers were invited to a deliberative forum. The deliberative forum method was initially developed to improve community involvement in health services decision making [21], but has more recently been adopted as an strategy to promote evidence-informed knowledge translation and policymaking [22]. The forum contained an educational component where the implementation models were presented and participants could question experts, followed by a deliberative section in which participants were asked to select a preferred model for the future implementation of home telehealth in South Australia.

The final stage of analysis developed the explanatory model and set of recommended implementation processes to support change management for future service development. This was done by adding the detailed contemporaneous notes from the forum, the FTH Trial evaluation reports, and relevant health policy documents to nVivo as source data. These were open coded and the thematic analysis already conducted on the interview data was extended to incorporate the new data. In regard to the grounded theory itself, an abductive analysis method [23], and a post-positivist epistemological approach [24] were chosen as a good fit for the research question. We hence assumed that our explanation is related to processes in the external world, although also recognised that the model would be one of many potential interpretations. In particular, we chose to focus on the selection of key influencing factors, as opposed to building a comprehensive list of all possible factors. This was done in order to be pragmatically useful to those who wish to initiate or promote change. The factors were then compared to the previously developed explanatory model of sustainable telehealth services (Fig. 1), looking for instances of support or contradiction as the new model was being constructed.

## Results

### Interviewee characteristics

Twenty individuals were approached to be interviewed; two declined and one offered a substitute, resulting in 19 interviews, which was the point at which data saturation occurred. 13 of the interviewees were health service managers, three were medical specialists and three were primarily concerned with service development. By sector affiliations, ten participants worked for government health services, four were from primary care, three from research organisations and two from aged care providers. All interviewees had senior positions within their own sectors, five being at the chief executive level, with the remainder leading either services or organisational units. In relation to the FTH trial, six participants were highly engaged, six were aware of its activities, and seven had minimal or no awareness.

### Preferred implementation approach

The first aim of this research was to develop a preferred governance and operational approach for a larger-scale home telehealth service. The analysis began with the portion of the semistructured interview transcripts in which participants were asked a set of questions on the practicalities of implementation. This material was brought together by the researchers into a small set of options and presented to the deliberative forum.

From the interviews, the participants showed great interest in these issues; several commented that there were no easy answers and most engaged in extended ‘thinking aloud’ about different options. Overall, there was strong support for wider implementation of home telehealth, however there was a diversity of views on the form of operations, with no one approach standing out. Taking each component in turn:

#### Governance

Responses reflected the complexity of the area, covering models of corporate ownership, who would take responsibility for clinical standards, and quality improvement. Five interviewees suggested government should take the lead in developing overarching governance of home telehealth, four that there should be a new organisation formed with strong community involvement, two wanted primary health networks to run the system, two preferred commercial companies, and one wanted an existing government-owned body to operate the service. Three proposed many models, and two interviewees did not answer the question in a classifiable way.

#### Funding models

The funding models proposed by interviewees were related to their options for operational management. When state government was suggested as the manager the funding mechanism would be block funding or activity-based funding, but interviewees said that a compelling argument for either hospital avoidance or service efficiency would need to be made to obtain such funds. Perverse incentives were reported, with a clinician noting that an increase in efficiency could lead to a reduction in one’s budget, and a manager saying, “we are constrained by the fear that we are going to create cost pressures inadvertently by allowing some sort of growth of activity that is not funded.”

In regard to private sector funding, some interviewees were in favour of the patient paying at least part of the cost, but it was recognised that the elderly or chronically ill were currently not paying out-of-pocket for care so this was not likely to be a viable or acceptable model. Grant funding was also regarded as possible, particularly for primary care organisations, but this did not lead to long-term financial stability for services.

#### Technical management

The need for high quality technical support to clinicians and patients was strongly emphasised, not only for present day services, but also to manage technology upgrades and new developments. The ability to deliver services using off-the-shelf devices was regarded as a strength of the FTH trial, however the back-end technical management of these devices was not highly visible and not as simple as it may have appeared to the participants. Interviewees regarded using the patient’s own device as the best option, for example, “in my house we have three iPads. I don’t want another iPad to be honest. I would be one of the people saying, just tell me what app I need.” Others pointed out that disadvantaged populations, such as those on limited incomes or with chronic mental illness, may need the health service to supply devices.

#### Models of care

Healthcare services operate according to explicit or implicit models of care, which set boundaries around the range of services, role delineation of providers, and type of patients with defined conditions or circumstances that will be accepted by the service. Interviewees noted that introducing home telehealth altered the model of care to being more structured; palliative care services changed from an environment where individual clinicians operated according to their own preferences, to a system with set criteria for data gathering and responses to patient status, and in aged care a structured nursing assessment was introduced before the telehealth consultation. Several interviewees considered that the model of care should be developed first, for example: “rather than jumping to a solution and getting a piece of technology as a hammer looking for a nail, build the model of care … then look at which technology solution or system solution would best fit supporting that.”

#### Data management

A wide variety of opinions existed about managing the information required and produced by home telehealth services, from the patient themselves, to general practices, primary care organisations, or central government IT services. Several interviewees said they could not give a definitive answer to this question.

From all of the above, it was clear that despite the interviewees’ seniority and experience in health services management, their views were underdeveloped, with little consistency in opinions about telehealth, even internally to each interview. It was common that an individual would recommend one structure for overall governance and a different structure for technical or data responsibilities. For example a service development manager put the view that a home telehealth organisation should be community-governed and not-for-profit, yet went on to say that the technical network should be operated by government health services.

The authors and other FTH trial team members discussed this material repeatedly until a set of future governance and operational options, together with their advantages and disadvantages, was constructed for presentation at the deliberative forum. This is summarised in Table 1. The options were categorised firstly into centralised versus distributed models, and then the centralised models were themselves divided into government-owned, private sector and consortium subgroups.

**Table 1:** Models for Home Telehealth Implementation

Model	Advantages	Disadvantages
1. Distributed Model Services are operated independently by each clinical unit or organisation.	Greater local control Easier to tailor to own needs	Cannot obtain economies of scale Difficulty with interoperability with external services Harder to scale up or down in response to changing demand Development needs to be done separately in each organisation, hence increased time needed to implement
2. Centralised Government Model State government provides all aspects: clinical services, technical network, device supply, management and IT support.	Small marginal cost to add home telehealth to an existing large ICT service Easy to scale up and down	A generic service may not suit all models of care Meeting privacy and security criteria may cause delays or abandonment of the service Restrictions on use of the service in the private sector
3. Centralised Commercial Model A commercial entity provides all technical services, and may also include clinical services.	Off-the-shelf products with more rapid implementation Easy to scale up and down Economies of scale for larger contracts	Less responsive to local needs May be limited to particular devices and systems Risk of higher-priced service contracts in a monopoly market
4. Centralised Consortium Model A group of providers forms a new not-for-profit entity.	Off-the-shelf products with more rapid implementation Providers have influence over the consortium Potential for the consortium to generate revenue and reduce costs for members The consortium can be a driver and innovator in the field	Time and effort required to build relationships, bring the partners together and construct agreements Potential conflict of members’ interests Members will initially have to fund central operations

#### The deliberative forum

The deliberative forum was an all-day event attended by 28 people, which included two expert presenters in health system funding and e-health service development. Most attendees were those who had been previously interviewed, but as the interview sample contained a minority of clinicians and none from general practice, intentional recruitment for the forum included two general practitioners who were active in policy and service development.

By the conclusion of the forum, a consensus was reached that the preferred option for larger-scale implementation was to form a collaborative consortium. Some attendees at the forum were willing to be members of such a consortium. There was caution about establishing a new organisation or written agreements immediately, with a preference to transition over a 12-month period to a formal arrangement. The forum recommended that implementation should be small scale at first, covering the continuation of the FTH trial services plus other projects that consortium members could contribute.

### The change management strategy model

**Fig. 2:**
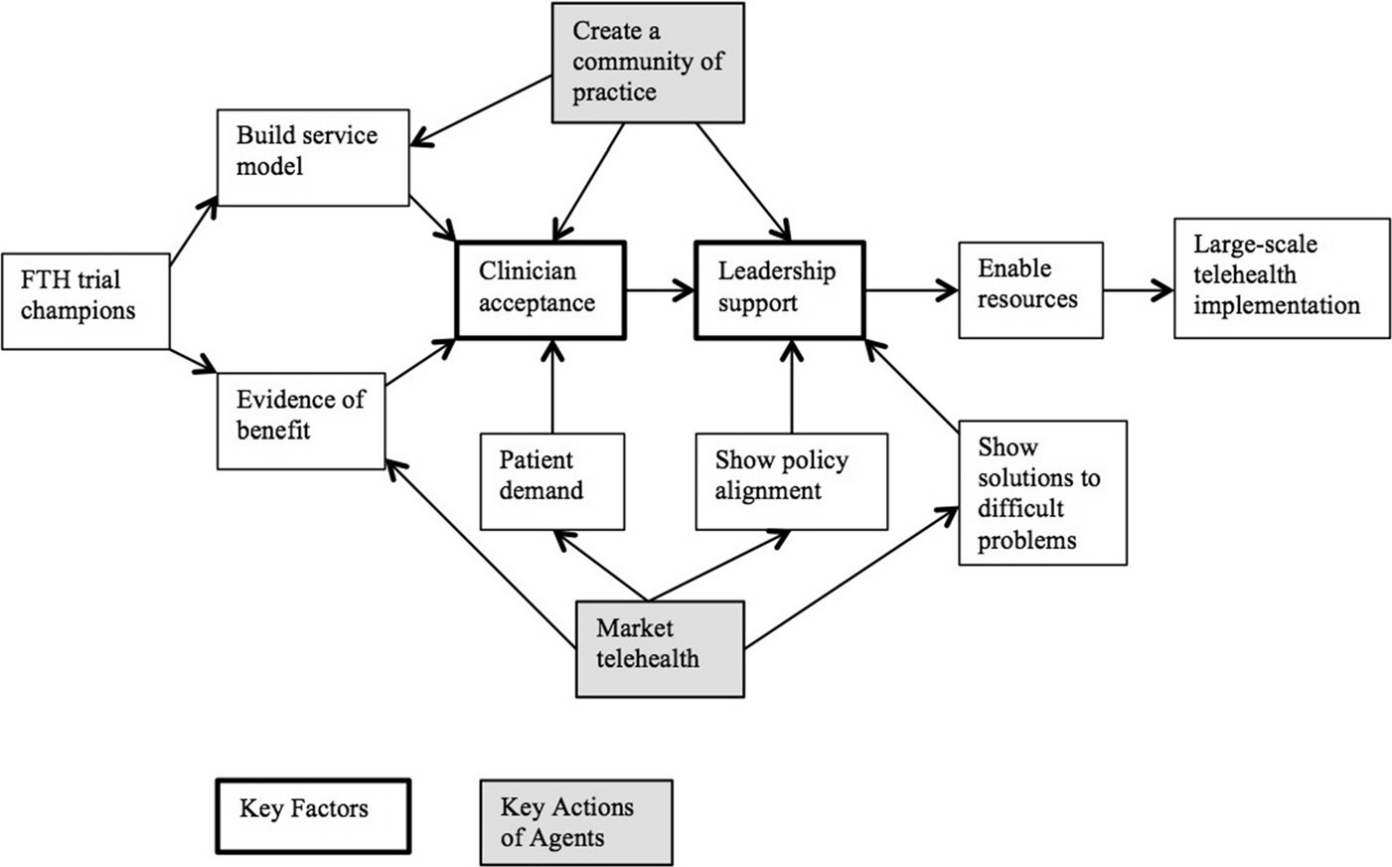
Model of Change Management Strategy for Large-Scale Telehealth

The second aim of the research was to develop a process model for assisting stakeholders in the change management needed to achieve broader implementation, and this is shown in Fig. 2. The sources of data used by the researchers in this endeavour were the prior model developed about the sustainability of telehealth services (Fig. 1), material from the semistructured interviews, FTH trial and policy documents, and the opinions from the deliberative forum. Whereas the prior model posited that clinician acceptance was the key factor to achieve sustainable telehealth services, this new model adds the second key factor of leadership support to achieve large-scale uptake. Each component of Fig 2 is now described.

#### Leadership support

Leadership support was regarded as a key factor because it was essential for allocating resources that are necessary for large-scale implementation, such as such as funding, workforce and equipment, plus giving operational permissions. The specific operational permissions described were allowing health service IT networks to connect to patients’ homes, counting telehealth as clinical activity, and approving budget allocations for new types of service. Interviewees particularly mentioned that differing perspectives between clinical services and IT services in the larger health system, plus the conservative nature of centralised IT departments, would be a problem for broader implementation. Although it was acknowledged that the FTH trial staff had worked hard to bridge this divide, it was stated that senior executives would need to support this approach in order for it to continue. The following factors described are those that lead into obtaining leadership support.

#### Show solutions to difficult problems

The ability to show how home telehealth could help with the problems faced by health leaders was considered by participants as an enabler for large-scale services. The problems were categorised thus:

i) Increasing Service Demand
  Interviewees noted increasing pressure on acute hospital services from two sources; a change in demographics to an older population with an increased burden of chronic disease, together with the inability of the health system to respond with new models of care. A senior manager held that, “over the past 20 years, it [healthcare system] has been talking about reducing hospital admissions and yet we still see this increasing demand curve. Something has got to change.”
ii) Budgetary Pressures
  During the course of the FTH trial, decisions taken at an Australian federal and state government level resulted in an environment of budgetary constraint. As a senior clinician put it bluntly, “in the current climate there will be no cases for nothing. Because there is no money around.” This meant, from the state government’s perspective, that would be no new funding for continuation of projects, or expansion of existing services, but new approaches that could demonstrate savings or efficiencies might be supported.
iii)  Service Delineation in Primary Care
  This problem concerns the relationship between hospital care and general practice.

In Australia, the federal government operates a national health insurance system that supports private specialist and generalist ambulatory medical care, and the state governments provide acute care and public hospital services, together with limited outpatient and community health services. Interviewees noted ongoing difficulties in achieving coordinated primary health care within this environment. A typical view was, “the State said it was Feds and the Feds said it was State so nobody ended up doing it”. This has been exacerbated by the state system pulling back from funding primary care services, as described by a senior manager, “State Health has been at pains to move their core business to acute services within hospitals and move away from hospital outreach or community based type services”. Introducing home telehealth was noted by hospital-based interviewees to provide an infrastructure that could better support coordinated chronic disease management. For example a clinician stated “the outreach is shared care with the GP but what it [home telehealth] is trying to do is to effectively provide both parties with more data, and to be able to provide the platform for more effective engagement with GPs.”

#### Show policy alignment

It is usual for health services to have a set of broad policies or principles, such as stated aims that healthcare should be accessible, timely, affordable, patient-centred, integrated, or cost-effective. For instance, the South Australian government describes key objectives as “improving coordination of care to ensure patients receive the right care in the right place at the right time” [25] Although the government has no extant policies about home telehealth, demonstrating that such a service would support these objectives was regarded as an enabler.

The opinions of the interviewees as to the importance of policy alignment varied from minor, “if the players are in the room saying how are we going to work together to achieve an outcome then it is actually better than a policy directive in my mind” to major, “I am not a policy person but I do recognise government policy is an important part of any business.”

#### Clinician acceptance

Clinician acceptance was the key factor in the prior model for achieving sustainability of telehealth services, and was regarded by participants as the third important influence on leadership support. From the interviews, those participants directly involved with the FTH trial expressed many positive comments about how the trial opened clinicians’ eyes to what is possible, and there was also high satisfaction from clinicians expressed throughout the trial evaluation. A cautionary note was sounded by some interviewees, however, indicating that clinician acceptance remained fragile: “I think clinician engagement is only just there. It hangs by a thread.” Probing as to why this might be the case brought out the following concerns:

i) Telehealth challenges existing models of care, and this change is not always welcome. Altering professional roles and workflow to adapt to these new approaches to practice was described as a substantial barrier. It also proved to be very difficult to engage GPs; reasons offered included GP concerns that consultant specialists were encroaching on their area, that it does not fit with the fee-for-service business model of private general practice, and that GPs are too busy and tired of change to participate.
ii) Several interviewees mentioned that lack of evidence for benefits was a problem, particularly for supporting large-scale uptake, eg “one of the issues is having sufficient data to say this is a change that should be service wide” and “we kept going to the State and saying we need money to keep this thing going … it is saving you a squillion in ambulance transfers. They just kept saying, prove it.”
iii)  Patient acceptability was important, in that participants thought clinicians would quickly abandon telehealth if it proved unacceptable to patients. A head of unit stated, “once [patients] are dissatisfied then clinicians go, well, we will go back to what we have always done, and you lose it [telehealth]”. Another senior clinician said, “if the patients see value then it is more likely that the clinicians will accept it.” Whereas active demand from patients could drive uptake, interviewees regarded patient awareness as not sufficient to produce action. An exemplar illustration of this point came from one clinician, “until we have got patients demanding it and saying ‘can’t I just talk to you over my iPad’, the clinicians probably have the sway in whether this is successful or not”.

### Enabling change

The final components added to the change strategy model were the ways in which a small group of people could best intervene to support change. Two key actions for enabling change were elucidated at the deliberative forum, which as well as selecting a preferred model of implementation, was also set the task of describing the means of achieving this.

#### Marketing telehealth

A multi-pronged approach to marketing was recommended as a means of overcoming resistance to change. There are three distinct audiences:

i) clinicians, who need to understand the new models of care developed by the FTH trial, and want evidence of benefit for patient access and outcomes.
ii) patients, to increase awareness of home telehealth and make it more likely they would enquire about this as an option for receiving services.
iii)  health service and political leaders, building the case for home telehealth contributing to management of difficult problems of demand and access, whilst demonstrating policy alignment.

#### Creating a community of practice

In healthcare, communities of practice have been proposed as a means of bringing together providers with common interests to solve problems and innovate [26]. Such communities can grow from simple discussion and dissemination of expertise to a more coherent group that shares resources or infrastructure. Communities of practice can serve as a means for engaging the wider clinical community (including general practitioners), supplying expertise in building telehealth service models, and becoming a consortium that could apply for further funding. A community of practice helps "normalize" telehealth, increasing confidence in the practice from clinicians and decision-makers. This can help overcome change fatigue and bridge the clinical-technical divide, providing both clinicians and IT staff are included.

## Discussion

One of the first lessons from this study was that, despite the participants’ experience in health services management, and within the FTH trial itself, study participants did not have fully formed views about how a larger telehealth service would be governed and operated. This suggests that the construction of large-scale telehealth services remains in the very early stages, in contradistinction to the marketing material emanating from industry forums and vendors.

As found in previous research [14], clinician acceptance continued to be an important enabler for the sustainability of telehealth services, but this research adds leadership support as the next key factor for wider uptake. Typically, telehealth begins as small services, started by champions who promote the method and also build relationships between providers [13]. In the expanded model in the FTH trial, where telehealth is intended to be normalised and expanded as routine practice, building individual relationships is replaced by building models of care that embed new institutional relationships and patient pathways.

While patient demand could be the primary driver of innovations in telehealth, our results suggest that clinician acceptance is the more important component and the literature supports this. For example, attempts to introduce shareable electronic records by first encouraging patients to enroll, and then hoping that health care providers and organisations will follow, have failed due to resistance of clinicians and healthcare organisations [27, 28].

Selecting the appropriate technology for the purpose is of great importance. On a cautionary note, multiple organisational barriers to IT projects in health have been noted [29], covering structures, policies, incentives, training, changes to work processes, and work culture issues. The relationship between clinicians and IT services can also be problematic, with the most frequent issues being that the internal IT departments of health service raise barriers to telehealth installations, place telehealth at the bottom of their list of competing priorities, and are reluctant to authorise non-standard systems [30]. The use of dedicated IT staff for the FTH trial ensured that these problems did not occur in this particular project, but they should be anticipated and managed during large-scale implementation

Using the patients’ own devices is in theory an enabler for broad scale uptake, given the high use of tablets and smart phones in Australia [31]. This would be difficult for some, such as individuals who are impoverished, have cognitive impairment, or are older with minimal digital literacy, and these are the very people who have more chronic conditions with a greater need for these types of health services. The FTH trial found it was necessary to provide the devices, the connectivity and the network management [32], so if health services want the benefits of home telehealth for these groups of people, they will need to incur these costs. This approach does have the advantages, however, that devices can be adapted for lower capabilities and limited to a small number of functions, plus reliable connectivity can be supplied.

The deliberative forum chose the consortium model as the preferred option for development. Successful examples are available from other jurisdictions, such as the Ontario Telemedicine Network, where a consortium established a not-for-profit organisation that became the dominant provider of telehealth infrastructure to most health care providers within one region [33]. This approach may not translate to the environment in Australia, which is more fragmented and competitive, with major stakeholders being less willing to contribute resources to collaborations. Nonetheless, the consultations undertaken during this study suggest that a small group, beginning with a community of practice, could make an impact. Such a community could only include a minority of clinicians, which is why moving to the normalisation stage supported by major stakeholders is so important.

The change management strategy model developed from the FTH trial can be generalised to similar circumstances where small groups of enthusiasts are attempting to introduce a complex change into a healthcare system. During times of plenty, there are resources to experiment with new systems and funding for trials, yet resistance often prevents real change on a broader level. At a time when health budgets are tight, it appears that the healthcare system in Australia has reached an impasse, in which it both cannot change and yet must change. Systems theory suggests that the more complex the system which contains a greater number of competing demands, the harder it will be to find a new optimum solution that will enhance the functioning of the whole system, hence the more resistance to change will occur. Coeira explicates how this applies in healthcare [34]. Telehealth in particular, is a complex intervention within an already complex environment, so resistance should be anticipated and managed. The model developed by this research offers focused advice for prioritising change management resource allocation.

Further studies of health system change in related environments would be helpful to assess the broader value of our model, which is limited by being based on one project, in one setting. Data collection took place over the short period of three months, and the participants themselves had not directly experienced the trial being transformed into a large-scale telehealth service. Strengths were, however, that this work was integrated into a longer term program of research into the uptake and implementation of telehealth, and that the participants were specifically chosen to be senior in status, experienced in health services development, and included those who would be directly responsible for large-scale implementation should this occur. Conducting qualitative studies on other telehealth service developments that are intended to become large-scale or have achieved this status, would add to the richness of the data, as would following up participants in our trial in one to two year’s time.

## Conclusions

A change management model for transitioning a home telehealth project from a trial into a routine service was built from the ground up. The main components of this model, suggested by qualitative data analysis, were new clinical and business models of care and evidence of benefits, which in turn supported clinician acceptance. Health service leadership support was the major factor needed to move trials to sustainable services and overcome resistances arising from lack of funds, change fatigue and the clinical-technical divide. The model proposes the use of change agents for marketing telehealth, demonstrating policy alignment and potential solutions for difficult problems, and the creation of aligned communities of practice supporting the case for change.

### Ethics statement

Ethics approval was granted by the Southern Adelaide Clinical Human Research Ethics Committee, application numbers HREC/13/SAC/88(168.3 and 203.13).

### Availability of data and materials

The original interview transcriptions cannot be made publically available due to the ethical requirement for de-identification of participants.

## Appendix

### Interview guide

About the project (for participants with direct involvement)

Could you outline what your role has been in this project?

What would you say are the main accomplishments of the project?

What have been the main challenges of the project?

Will your organisation be taking any role in the continuation of project activities services past Sept 30?

Will there be any continuing research and/or evaluation?

What future developments would you propose that your organisation be involved in?

How would you see a broader rollout of home telehealth occurring in South Australia? What do you think it should look like?

What do you see as the challenges and barriers of rolling out telehealth in South Australia?

### About the project (for participants without direct involvement)

Have you had any involvement with or awareness of the home telehealth project?

Can you give me an outline of your work role?

If some knowledge of the project, what are your thoughts on how the project has been going to date? Any thoughts in particular on how this changes the delivery of healthcare or the model of care?

Topic prompts on specific issues

*Governance*:  who should operate home telehealth services? What could or should be the role of the following groups?

SA Health

Medicare Locals or PHNs

General Practices

Commercial companies ie Telcos, health informatics groups.

The not-for-profit sector

A new entity formed; could (your organisation) be part of that?

*Data Handling*

Home telehealth generates new data. Who should collect it and be responsible for its management? How should this relate to other clinical data systems?

Technical matters issues to do with the telehealth equipment or telecommunications; should the service loan out equipment or use the patient’s own infrastructure?

*Funding*,  ie sources of revenue or budget for the service.

What sort of business model do you think would work for the service?

Can you identify any actual savings from the use of telehealth?

What about the possible use of Medicare item numbers?

*Three models to consider*

- SA Health pays Telehealth in the Home ICT services as a public service, and offsets the cost against reduced costs in the health system eg hospital admissions, more efficient use of staff time and so forth

- User pays; the patient is charged a fee

- Subscription basis; each health care organisation (including SA Health) pays an annual subscription for use of Telehealth in the Home ICT services

*Clinical issues*, ie the ability to deliver the service using telehealth, the quality of service

*Acceptability* to clinicians; their attitudes to telehealth. Patient issues: do you think older patients are accepting of telehealth? Are there any patient barriers?

*Organisational factors*, ie workforce, job roles, structure of the service, new service models

*Policy environment*, ie presence or absence of policies about telehealth

*Political issues*, either at the local level or in the larger environment

*Ethical issues*, such as privacy, confidentiality

*Legal issues*, eg indemnity, professional registration

*Research issues*, such as results of the evaluation of the service

Any other factors not yet mentioned?

Interview Conclusion

- Do you have any other comments?
- If I have some additional questions to ask you at a later date, would you be willing to be contacted again? Whether or not you choose to participate in a second interview would be entirely your choice at the time.
- Would you like to receive information about the outcomes of this study?

Next Steps

- Would you be interested in/willing to attend a forum around August at which practical models for sustainability would be presented and discussed?

## Abbreviations

FTH Trial: Flinders University telehealth in the home palliative and aged care trial
RCT: randomised controlled trial
IT: information technology
GP: general practitioner

## References

[1] Munro J, Angus N, Leslie SJ (2013). Patient focused internet-based approaches to cardiovascular rehabilitation - a systematic review. J Telemed Telecare.

[2] Bradford NK, Armfield NR, young J, Smith AC: The case for home based telehealth in pediatric palliative care: a systematic review. BMC Palliative Care 2013:12(4). doi:10.1186/1472-684X-12-4. 10.1186/1472-684X-12-4 PMC3584741 23374676

[3] Crotty M, Killington M, van der Burgh M, Morris C, Taylor A, Carati C (2014). Telerehabilitation for older people using off-the-shelf applications: acceptability and feasibility. J Telemed Telecare.

[4] Tieman J, Morgan DD, Swetenham K, To TMT, Currow DC (2014). Designing clinically valuable telehealth resources: processes to develop a community-based palliative care prototype. J Med Internet Res.

[5] Merrell RC, Doarn CR (2012). Editorial: barriers or barricades. Telemed J E Health.

[6] Miller EA (2007). Solving the disjuncture between research and practice: telehealth trends in the 21st century. Health Policy.

[7] Broens TH, Veld RM H i't, Vollenbroek-Hutten MM, Hermens HJ, van Halteren AT, Nieuwenhuis LJ (2007). Determinants of successful telemedicine implementations: a literature study. J Telemed Telecare.

[8] Jarvis-Selinger S, Chan E, Payne R, Plohman K, Ho K (2008). Clinical telehealth across the disciplines: lessons learned. Telemed J E Health.

[9] Obstfelder A, Engeseth K, Wynn R (2007). Characteristics of successfully implemented telemedical applications. Implement Sci.

[10] Robert G, Greenhalgh T, MacFarlane F, Peacock R (2010). Adopting and assimilating new non-pharmacological technologies into health care: a systematic review. J Health Serv Res Policy.

[11] Cresswell K, Sheikh A (2013). Organizational issues in the implementation and adoption of health information technology innovations: an interpretive review. Int J Med Inform.

[12] May C, Harrison R, Finch T, MacFarlane A, Mair F, Wallace P (2003). Understanding the normalization of telemedicine services through qualitative evaluation. J Am Med Inform Assoc.

[13] Wade V, Eliott J (2012). The role of the champion in telehealth service development: a qualitative analysis. J Telemed Telecare.

[14] Wade VA, Eliott JA, Hiller JE (2014). Clinician acceptance is the key factor for sustainable telehealth services. Qual Health Res.

[15] Wade V, Eliott J, Karnon J, Elshaugh AG (2010). A qualitative study of sustainability and vulnerability in Australian telehealth services. Stud Health Technol Inform.

[16] Hendy J, Chrysanthaki T, Barlow J, Knapp M, Rogers A, Sanders C, Bower P, Bowen R, Fitzpatrick R, Bardsley M (2012). An organisational analysis of the implementation of telecare and telehealth: the whole systems demonstrator. BMC Health Serv Res.

[17] Darkins A (2014). The growth of telehealth services in the Veterans Health Administration between 1994 and 2014: a study in the diffusion of innovation. Telemed J E Health.

[18] Waterman H, Marshall M, Noble J, Davies H, Walshe K, Sheaff R, Elwyn G (2007). The role of action research in the investigation and diffusion of innovations in health care: the PRIDE project. Qual Health Res.

[19] Birks M, Mills J (2011). Grounded theory: a practical guide.

[20] QSR International: NVivo In., 8 edn: QSR International; 2009: Computer software.

[21] Abelson J, Forest P-G, Eyles J, Smith P, Martin E, Gauvin F-P (2003). Deliberations about deliberative methods: issues in the design and evaluation of public participation processes. Soc Sci Med.

[22] Boyko JA, Lavis JN, Abelson J, Dobbins M, Carter N (2012). Deliberative dialogues as a mechanism for knowledge translation and exchange in health systems decision-making. Soc Sci Med.

[23] Kelle U (2005). "Emergence" vs. "forcing" of empirical data? A crucial problem of "Grounded Theory" reconsidered. Forum.

[24] Annells M (1996). Grounded theory method: philosophical perspectives, paradigm of enquiry, and postmodernism. Qual Health Res.

[25] Primary Health Networks [http://www.health.gov.au/internet/main/publishing.nsf/content/primary_health_networks].

[26] Ranmuthugala G, Plumb JJ, Cunningham FC, Georgiou A, Westbrook JI, Braithwaite J (2011). How and why are communities of practice established in the healthcare sector? A systematic review of the literature. BMC Health Serv Res.

[27] Greenhalgh T, Hinder S, Stramer K, Bratan T (2010). Adoption, non-adoption, and abandonment of a personal electronic health record: case study of HealthSpace. Br Med J.

[28] Kidd MR (2008). Personal electronic health records: MySpace or HealthSpace?. Br Med J.

[29] Lluch M (2011). Healthcare professionals’ organisational barriers to health information technologies - a literature review. Int J Med Inform.

[30] Wade R, Cartwright C, Shaw K (2012). Factors relating to home telehealth acceptance and usage compliance. Risk Mgmt Healthcare Policy.

[31] Communications Report 2011–12 Series; Report 3 - Smartphones and tablets take-up and use in Australia [http://www.acma.gov.au/webwr/_assets/main/lib310665/report-3-smartphones_tablets-summary.pdf].

[32] Taylor A, Wade V, Morris G, Pech J, Rechter S, Kidd M, Carati C. Technology support to a telehealth in the home service: qualitative observations. J Telemed Telecare 2015:1357633X15601523. 10.1177/1357633X15601523 26362562

[33] Brown EM (2013). The Ontario telemedicine network: a case report. Telemed J E Health.

[34] Coiera E (2011). Why system inertia makes health reform so difficult. BMJ.

